# Beyond beauty: A qualitative exploration of authenticity and its impacts on Chinese consumers' purchase intention in live commerce

**DOI:** 10.3389/fpsyg.2022.944607

**Published:** 2022-09-09

**Authors:** Jiani Sun, Honorine Dushime, Anding Zhu

**Affiliations:** ^1^Modern Business Research Center, Key Research Institute of Humanities and Social Sciences for Universities, Zhejiang Gongshang University, Ministry of Education of China, Hangzhou, China; ^2^School of Management and E-Business, Zhejiang Gonghang University, Hangzhou, China

**Keywords:** live commerce, live streaming video, live shopping, authenticity, in-depth interview, qualitative approach, grounded theory

## Abstract

Live commerce is a phenomenally innovative form of social commerce in China. In this paper, the authors aim to explore the authenticity of live commerce. By employing a qualitative approach using in-depth interviews and grounded theory, 21 initial categories are classified into six core categories. Among them, authenticity-associated concepts are classified into explicit concepts and implicit concepts. Explicit concepts of authenticity are associated with objectively authentic cues, while implicit concepts of authenticity are associated with subjectively authentic experiences. Moreover, the study explores the relationship between explicit concepts of authenticity and product commitment, as well as the relationship between implicit concepts of authenticity and affective commitment. Both of these paths are found to influence consumers' shopping-related behaviors. Although consumers can more easily perceive explicitly authentic cues than implicitly authentic experiences, this study suggests that the latter may be more effective in inducing shopping behaviors. In addition, the effect of streamer attractiveness on opinion leader building is addressed, while authenticity is found to be an alternative approach to attract consumers both for attractive and nonattractive streamers. Finally, the study addresses theoretical implications and practical implications as well as suggestions for future research.

## 1. Introduction

Live commerce is a phenomenally innovative form of social commerce in China. Live commerce has become increasingly popular in China's online market since Alibaba initiated Taobao Live in May 2016 (Taobao Inc., 2020). Compared with traditional e-commerce, live commerce allows streamers to demonstrate products and interact with consumers *via* live streaming video in real time. According to the 47th survey report of the China Internet Network Information Center (CNNIC), the user size of live streaming in China had reached 703 million as of December 2021, accounting for 68.2% of all Internet users. Meanwhile, the user size of live commerce in China had reached 464 million, accounting for 44.9% of all Internet users. The CNNIC's report summarized three reasons for the rapid development of live commerce in China: (1) local governments encourage the development of the industry; (2) active enterprises provide technical support for the development of the industry; and (3) extensive participation of Internet users accelerates the development of the industry (CNNIC, 2022). China's live commerce industry has reached $348 billion as of December 2021; and is estimated to $515 billion in 2022 (100EC.cn, 2022). On Taobao's 2021 annual Single-Day Shopping Festival (SDSF), live commerce accounted for $10.8 billion in sales (AskCI.com, 2021). This trend has also spread to the United States and other countries. Amazon has also launched its live platform (https://www.amazon.com/live), while Facebook and Instagram are trying to allow users to browse products and place orders directly within their social media (Pan et al., 2022).

Live commerce allows celebrities, also known as influencers, streamers, or presenters, to sell products and services *via* online live video streaming. The live streamer is the only main character in the center of the live streaming stage. In the majority of the cases, a steamer will be more successful if (s)he is more beautiful or attractive. Physical attractiveness was found to correlate positively with happiness and self-esteem, especially for women (Mathes and Kahn, 1975), which implies a beautiful streamer will be more self-confident and willing to become a live celebrity. O'Connor and Gladstone (2018) verified that more attractive people would be more likely to select more profitable broker positions and acquire more social capital for themselves. Moreover, noted economist Daniel Hamermesh demonstrated that better-looking people experience undeniable benefits in all aspects of life (e.g., getting better jobs, working more productively and profitably, receiving more substantial pay, and negotiating loans with better terms) (Hamermesh, 2013). People instinctively trust and prefer information from more attractive informants (Bascandziev and Harris, 2014), who are more likely to become opinion leaders or celebrities (Du et al., 2022). Slaton (2013) revealed that the attractiveness of models lowers consumers' self-esteem and increases their purchase intention from the perspective of social comparison.

On the other hand, another literature stream tries to challenge the “beauty-is-good” aphorism. Griffin and Langlois argued that although unattractiveness is a disadvantage, attractiveness is not as advantageous as imagined (Griffin and Langlois, 2006). Others have tried to distinguish trustworthiness from attractiveness. Ert et al. (2016) reported that more trustworthy-looking Airbnb hosts charge higher prices for apartments than more attractive-looking hosts from Stockholm. The result was challenged by Jaeger et al. (2019), who investigated 1,020 listings in New York City and found that more attractive-looking hosts charge higher prices rather than more trustworthy-looking hosts. Reputation and expertise are other alternatives of attractiveness in the celebrity literature. Zhou and Whitla (2013) reported that Asian consumers negatively react to the poorly behaving celebrities and their endorsed brands. Van Norel et al. (2014) revealed that an intelligent celebrity who has the best fit with a topic will restore a positive public opinion about a damaged corporate reputation through his or her tweets.

Many live commerce streamers are not attractive in the real world. Some of them are even rustic: they live in the mountains, wearing low-quality clothes, and present their original lifestyle. Especially during the COVID-19 lockdown, a large number of agricultural products encountered the dilemma of being unsalable (Zuo et al., 2022b). Furthermore, supply chains were also vulnerable because of demand fluctuation (Liu et al., 2022). Live commerce provided an alternative channel opportunity to promote products straightly to end consumers. Our question was what factors attract Chinese consumers to participate in and purchase products in live shopping endorsed by unattractive streamers. Why do Chinese consumers trust unattractive streamers?

An increasing body of live commerce literature provides some clues to answer the above questions. As a new method of influencing consumer purchase, live commerce is a sub-class of s-commerce, integrating real-time interactivity with e-commerce (Kang et al., 2021). The interactivity leverages value cocreation between streamers and consumers, affecting consumers' mentally simulated experiences, and in turn affecting their intention to continue using a live streaming service (Chou et al., 2022). Deng et al. (2022) argued that live streaming affordances provide opportunities to foster parasocial relationships between streamers and consumers through psychological, social, and technological dimensions, developing parasocial acquaintances. For Chinese consumers, the social bonds (Hu and Chaudhry, 2020; Zhou et al., 2021), also known as “guanxi” (Guo et al., 2021b) in Chinese social context, have proven to be positively related with consumer trust on engagement in live streaming commerce. Furthermore, Lo et al. (2022) revealed that parasocial interaction contributes to consumers' affective reactions, leading to their impulsive buying behaviors. Especially during COVID-19 containments, consumers' purchase intention was influenced by their feelings of loneliness and boredom (Peng et al., 2022; Zuo et al., 2022a), and live commerce provided emotional support and social presence (Chen and Liao, 2022).

Although streamer attractiveness was found to play a significantly moderating role between consumers' social presence and live streaming engagement (Pan et al., 2021; Chen and Liao, 2022) regarded homogenization of live content as one of the existing problems of live streaming commerce. Extant studies suggested that streamer genre (Zhang et al., 2022a), entertainment perception (Chen et al., 2020a), and information credibility (Wang et al., 2022) can facilitate streamers to implement differentiated competition strategies. In the present study, the authors attempted to explore the impact of authenticity of live streaming commerce on Chinese consumers' purchase behavior because we identified three kinds of authenticities (i.e., streamer authenticity, product authenticity, and scene authenticity) from three typical examples of Chinese live streamers (i.e., Denggao Chen, Viya, and *Peach Sister in Sichuan*). Authenticity has been an issue for tourism studies for nearly 50 years, ever since MacCannell introduced this museum-related concept into sociological studies of tourist experiences (MacCannell, 1973). Authenticity was found to be one crucial determinant for tourists to perceive and to experience cultural destinations (Boukas, 2012). Wang (1999) classified authenticity into two categories, i.e., object authenticity and existential authenticity. The authors also attempted to explore the authenticity classifications in the context of live commerce. Live commerce is a mixture of telecommunication and commerce. Many studies have confirmed that authenticity is highly associated with tourists' shopping behavior (Fu et al., 2018; Liang et al., 2018; Kim and Kim, 2020). However, to the best of our knowledge, it remains unclear how consumers experience authenticity and how authenticity subsequently affects consumers' purchase intention in live commerce. To bridge the knowledge gap, a qualitative approach and the grounded theory were employed in the present study. Twenty-three interviews were conducted in total before achieving the theoretical saturation. Authenticity-associated concepts were classified into explicit concepts and implicit concepts. Twenty-one initial categories were classified into six core categories. Among them, explicit concepts of authenticity were associated with objectively authentic cues, while implicit concepts of authenticity were associated with subjectively authentic experiences. This study contributes to the literature of both live commerce and authenticity. Furthermore, this study also benefits live commerce practitioners by exploring authentic resources to implement differentiated competition strategies.

## 2. Literature review

### 2.1. Authenticity

Authenticity was originally used in the museum-related issues to clarify “whether objects of art are what they appear to be or are claimed to be” and was then borrowed to refer to “human existence and anxiety over the credibility of existence” (Trilling, 1972). MacCannell (1973) adapted this concept to explain tourist experiences. In the context of tourism, “authenticity connotes traditional culture and origin, a sense of the genuine, the real or the unique” (Sharpley, 1994). MacCannell (1973) claimed that tourists desire authentic experiences because of the fulfillment of escaping to other places and times. Wang (1999) classified authenticity into two categories: object authenticity and existential authenticity. He suggested that both the toured objects and the authentic experience can coexist. He argued that tourists engage in camping, walking, or wilderness solitude because they search for their authentic selves rather than toured objects. Alternatively, Selwyn (1996) associated the experience of a “real” world with “cool” authenticity and the experience of a “real” self with “hot” authenticity. Currently, the concept of authenticity has become more diversified. In the emerging accommodation sharing sector, authenticity refers to seeking local living experiences for Airbnb consumers. Mody and Hanks (2019) expanded three dimensions of brand, existential, and intrapersonal authenticity in creating brand-loving and brand-loyal customers for Airbnb accommodation. Aykol et al. (2017) expanded the relationship between the authenticity of core and peripheral aspects of the arts experience. In addition, Baker and Kim (2018) argued that language and ethnic appearance also affect consumers' perceptions of authenticity.

Extant studies have revealed a high correlation between authenticity perception and consumer purchase intention. In the conventional tourism sector, many studies confirmed that perceived authenticity plays a crucially positive role when tourists make their decisions to buy souvenirs (Fu et al., 2018), book and rebook Airbnb rooms (Liang et al., 2018), and purchase organic foods (Lu et al., 2019). In regard to online shopping, authenticity influences consumer behaviors from three aspects: telepresence, differentiation, and trustworthiness. In the era of mobile commerce, innovative communication tools construct an online virtual environment to enhance consumers' telepresence perception (Cohen, 2011). Meanwhile, social presence and telepresence narrow the psychological distance between sellers and buyers, creating online authenticity and empathy (Ou et al., 2014). Second, authenticity is a tool used to create differentiated competitive advantages for sellers (Aykol et al., 2017). Finally, authenticity is often reported to be associated with sellers' trustworthiness, which will subsequently impact consumers' decision-making (Zatori et al., 2018; Shuqair et al., 2019).

In China, perceived authenticity was found to have a significant effect in reducing Airbnb consumers' perceived risk and positively influencing their perceived value (Liang et al., 2018). Kim and Kim (2020)'s empirical study also indicated the significant roles of perceived authenticity of online comments and trust in the context of online tourism. Safeer et al. (2021) revealed that perceived brand authenticity dimensions significantly impacted brand love, which positively affected Asian millennials' behavioral outcomes. Additionally, Chinese e-retailers are always willing to try new digital marketing strategies to provide more authentic information to their customers. Peng and Ke (2015) noted that the three-dimensional (3D) virtual technology provides a new promotion channel to increase users' perceptions of authenticity and trustworthiness. Zhang et al. (2020) proved that the live video streaming (LVS) strategy can improve customers' online purchase intention by providing more authentic information to reduce psychological distance and uncertainty. Guo et al. (2021a) and Baek et al. (2020) confirmed the positive effect of live streaming features on reducing consumers' overall perceived uncertainty, and increasing consumers' overall perceived value and purchase intention in the context of cross-border e-commerce.

### 2.2. Live commerce

As an emerging phenomenon, live commence has been attracting more and more attention in recent years. Extant studies of live commerce mainly include three aspects: the technological issues of live streaming systems, the social commerce perspective, and relevant consumer behaviors. Sun et al. (2019) surveyed live-streaming shopping platforms, e.g., Taobao.com, JD.com, Mogujie.com, and Sina Microblog and found that IT affordance, including visibility affordance, metavoicing affordance, and guidance shopping affordance, is positively associated with consumer engagement. Choi and Jeon (2022) also confirmed the positive effect of IT affordance in South Korea, of which trading affordance has the most significant effect. They suggested the live commerce platform to devise a program that helps make payment easier for users. Yang et al. (2022b) identified three sales modes of live streaming commerce (i.e., e-commerce platform mode, transferring mode and live streaming platform mode) and suggested that hybrid mode may generate higher profits for members, except for the live streaming platform. Additionally, Zhang et al. (2022a) revealed that trust can be enhanced through live interactivity and technical enablers (e.g., visibility and personalization) in live commerce. Xie et al. (2022) regarded live streaming as a technology-enabled and value-based tourism marketing tool.

Second, to figure out how live streaming video enhances the social relationship in social commerce, Hu and Chaudhry (2020) identified three relational bonds between the broadcaster and the consumers, i.e., financial bonds, social bonds, and structural bonds. They verified that relational bonds are highly associated with consumers' affective commitment to the broadcaster and online marketplace, subsequently predicting consumers' engagement behavior. Chen et al. (2020b) investigated the prosocial interaction experience between service providers and live streaming users. They developed and validated an Online Live Streaming Perceived Servicescape (OLSPS) scale to improve user satisfaction. Kang et al. (2021) and Guo et al. (2021b) revealed interactivity plays a mediating role between relationship and consumer behavior. Wongkitrungrueng et al. (2020) observed that Facebook users have a greater range and dispersion of engagement metrics (e.g., comment, share, and reactions) for videos than for photos, statuses, and links. They identify four live streaming sales approaches: the transaction-based approach, persuasion-based approach, content-based approach, and relationship-based approach, which leverage customers' trust in products and sellers to engage in live streaming sales (Wongkitrungrueng and Assarut, 2020). Likely, Chandrruangphen et al. (2022) confirmed that live streaming sellers can develop trust with their customers because price transparency. Additionally, Yang et al. (2022a) identified six categories of cocreation patterns in China's live-streaming travel industry.

Third, scholars attempted to understand consumers' shopping behaviors associated with live commerce. Lu and Chen (2021) noted that live streaming reduce product uncertainty and cultivate trust for the consumers, facilitating their purchase intention. Zhang et al. (2020) examined the construal level of live streaming and demonstrated its effects on reducing consumers' psychological distance and perceived uncertainty, which in turn increases consumers' online purchase intention. However, Zhou et al. (2021) argued that the purchase intention is influenced by age, gender, education, and income. Wang et al. (2021) revealed consumers' flow experience in live streaming is positively affected by streamers' charm and interaction. Likely, Xu et al. (2020) confirmed that streamer attractiveness and parasocial interaction are antecedents of consumers' live shopping behaviors, e.g., hedonic consumption and impulsive consumption. In fact, Lee and Chen (2021), Zhang et al. (2022b), and Ming et al. (2021) demonstrated that consumers make impulsive purchase decision because of urging of the liver streamer in a short period, social presence of live streamers, and consumers' sense of power. On the other hand, the streamers' persuasiveness (Gao et al., 2021), humor appeal and sex appeal (Hou et al., 2020) also positively impact consumers' watching intention and purchase intention. In addition, studies from different Asian countries (e.g., South Korea Lee and Kwon, 2022, Thailand Chandrruangphen et al., 2022, and Malaysia Ong et al., 2021) have also confirmed similar conclusions.

### 2.3. Celebrity

A celebrity is a person or group who enjoys fame and has broad public recognition in mass media. A celebrity can establish active parasocial relationships with fans through personal self-disclosure (e.g., sharing their friends and family) and professional self-disclosure (e.g., sharing their work-related life) (Kim and Song, 2016). Han and Ki (2010) found that celebrity reputation represents six dimensions, i.e., personalities, relationships, appearance, expert abilities, management of their private lives, and reputation. For many years, corporations have learned how to use celebrities' reputations to promote credibility (Hussain et al., 2021). Goldsmith et al. (2000) reported that celebrity credibility has the strongest impact on consumers' attitudes toward the ad, while corporate credibility has the strongest impact on consumers' attitudes toward the brand. Ambroise et al. (2014) moved one more step further; they proposed that a corporate brand can take advantage of a celebrity spokesperson's personality to create a cobranding personality. Reputation-damaged corporations also employ celebrities to repair their reputations (Van Norel et al., 2014).

Beauty or attractiveness is one of the crucial personal traits for an Internet celebrity, but not in general (Mathes and Kahn, 1975; Hamermesh, 2013; O'Connor and Gladstone, 2018). Park and Lin (2020) found that celebrity trust is based on a celebrity's optimistic characteristics and/or goodwill toward consumers. The match-ups of celebrity-product fit, live content-product fit, and self-product fit are positively associated with consumers' attitudes and purchase intentions. In fact, early in the 1990s, Ohanian (1990) tried to distinguish celebrity endorsers' characteristics of perceived expertise, trustworthiness, and attractiveness and their effects on consumers' behavioral intentions. Geng et al. (2020) captured both first-order effects and second-order effects of Internet celebrity endorsements on marketing outcomes in an e-commerce context. They classified the content generation and interaction behaviors between marketers and consumers as the first-order effects of Internet celebrity endorsement and interactions within the fan community as the second-order effects. Cuomo et al. (2019) explored the relationships among celebrity credibility, celebrity familiarity, luxury brand value, and brand sustainability awareness on attitudes toward celebrities, brands, and purchase intentions for sustainable consumption.

## 3. Case study

In this study, the authors first identified three object authenticities in live commerce (Reisinger and Steiner, 2006), i.e., streamer authenticity, product authenticity, and scene authenticity. Online live streaming video narrows the psychological distance between streamers and their audience by bringing the audience into an authentic environment.

### 3.1. Case 1: Streamer authenticity

After the lifting of the national-wide COVID-19 lockdown, most Chinese local governments worked hard to promote local agricultural products. As an example, on May 6, 2020, Denggao Chen, the mayor of Yanglin Town, Yueyang County, Hunan Province, presented live streaming, in which 2,500 locally organic products were sold out in 90 min (Hunan, 2020). The government official was not a good-looking man. Instead, he looked like a farmer. However, the performance was better than expected. The product he endorsed was an improved pomelo variety that was produced organically in the local hills.

The authenticity was the combination of the streamer's appearance, accent, and discourse. The streamer told the audience that he had grown up and was still a resident of the town. The news presented the streamer who had a simple and grounded country appearance with a dark face, which was similar to that of ordinary local farmers. The streamer knew the local culture and the local special products well. The news acclaimed that he knew how to convey the product's characteristics and merits to an audience in a short time and understood how to embed the product into the local customs and traditions. In addition, the streamer did not have a good command of standard pronunciation and mixed a few dialects and slang from time to time, which increased the audience's perception of his authenticity and trustworthiness.

### 3.2. Case 2: Product authenticity

Weiya (Viya) is the most popular live-streaming celebrity in China. She has been contributing to charity since 2016 by live promoting agricultural products for villagers and farmers from the central and western provinces of China (China Daily, 2021). To maintain her authenticity, she always chooses to live stream in the county of origin. For example, she sold Anhui's special food, *Ligao*, in Anhui Province, Qinhai's intangible cultural heritage, *Qingxiu*, in Qinhai Province, and Yunnan's pomegranates in Yunnan Province. Product authenticity is a combination of the layout with local culture, the demonstration of the products at the fields and farms, and the interaction between farmers and products. The streamer tried to rebuild the environment where the products exist. When the products leave their original environment, the information attached to the products will disappear. Consumers prefer standardized products with a good appearance. However, to remain in a good condition, most products need to be handled technically. The live streaming video presents the product with its original information, helping consumers identify the original shape, color, and appearance.

### 3.3. Case 3: Scene authenticity

Some live streamers choose the live streaming rooms in the real-life scenes. They use live streaming video to record their daily lives. Their lifestyles may be quite different from those of the audience. For example, a famous celebrity, whose nickname is *Peach Sister in Sichuan*, established her live streaming room in her kitchen (YNET.cn, 2021). Her secret seasoning, well-known as *BoBo Chicken Seasoning*, has established a delicious food reverie for millions of audiences. Her home is an ordinary and primitive village house that is even a little dilapidated. Her kitchen is in a tough condition. However, the audience recognizes her simple and authentic lifestyle that they may have experienced when they are young. The scene authenticity establishes the relationship between the streamer and the audience through time and space.

## 4. Data and methodology

### 4.1. Data

To explore audience perceptions of authenticity and their effects on their purchase intentions, several in-depth interviews were conducted. Because live commerce is more popular and acceptable in China, we recruited only Chinese live commerce participants and shoppers. The interviews were conducted in the Chinese language. The concept of authenticity was translated into Chinese terms, which can be easily understood by ordinary Chinese informants. Douyin, also known as TikTok outside China, is one of the top three live commerce platforms in China. According to its 2020 report, users aged 22–35 account for approximately 40% of its 600 million daily active users (DAUs) (Douyin.com, 2020). Moreover, the proportion of female users (55%) is slightly higher than that of male users (45%). The informants were recruited by employing convenience sampling technology (Maxwell, 2012). There were sufficient potential informants because of the wide population coverage of Douyin's live commerce. Hence, we filtered college students and young company employees who had shopped *via* Douyin live commerce at least three times. To make informants understand so-called “authentic” scenes during live shopping, we demonstrated the three cases above to help them recall their similar shopping experiences. The final selected informants all confirmed at least one similar shopping experience. Table 1 lists the descriptive statistics of the informants.

**Table 1:** The descriptive statistics of the informants (*N* = 23).

**Variables**	**Categories**	**Frequency**	**%**
Gender	Male	10	43.5
	Female	13	56.5
Age	Less than 18	0	0.0
	18-28	18	78.3
	Larger than 28	5	21.7
Educational Level	Senior high school and below	0	0.0
	Bachelor degree	19	82.6
	Master degree and above	4	17.4
Occupation	Student	18	78.3
	Unemployed	0	0.0
	Employed	5	21.7
Have you purchased items promoted by live streaming at least	Yes	23	100.0
three times?	No	0	0.0
Do you have at least one similar shopping experience to the	Yes	23	100.0
three cases?	No	0	0.0

### 4.2. Methodology

#### 4.2.1. Rationale

The approach of grounded theory (GT) was chosen for a number of reasons to explore the authenticity concepts in live commerce (Glaser and Strauss, 1967; Strauss and Corbin, 1998), such as the following: (1) live commerce is now an emergent e-commerce mode. There is still little knowledge about the authenticity in the context of live commerce in the literature; (2) live commerce is also a fast-growing sector, which has various forms and contents. At present, a lot of research is needed to collect the authenticity nature of live commerce; and (3) There is still a lack of authenticity scales or instruments for conducting quantitative researches. Therefore, there is a need for appropriate and rigorous qualitative investigations to explore and identify a set of authenticity concepts in live commerce. The ground theory approach is a suitable option (Mahrous and Kortam, 2012).

#### 4.2.2. Data collection

The data were collected by in-depth interviews. First, a semi-structured interview guide with open-ended items based on the literature review and case studies was created. The main focus of the semi-structured interview guide was to increase reliability and explore the informants' authenticity perception and motivation for their shopping decision-making while participating in the live commerce activities. According to the section of case study, initial concepts of authenticity was identified as the roots.

All these three cases inspire us to conclude that the live commerce can take advantage of live streaming video to increase audiences' perceptions of object authenticity. The authenticity presented through live-streaming videos contains explicit and implicit concepts. Explicit concepts refer to the authentic features associated with the streamer, the product, and the scene, which can be identified and observed by the audience. Explicit concepts convey concrete and representational information to the audience, motivating them to make judgments and purchase decisions. For example, the appearance, the dialect, and the accent of the official streamer demonstrate the association of explicit information with the local environment.

From the theory of authenticity, in addition to object authenticity, existential authenticity can explain a greater variety of tourist experiences (Wang, 1999). Live streamers are used to induce audiences to substitute themselves into specific scenes. Hence, implicit concepts refer to the cues, hints, and metaphors that can be interpreted by the audience to establish simulations and telepresence, e.g., the consumption scenes, the application scenes, and offline shopping scenes. The suggestive scenes may be conveyed through descriptive commentaries, cultural inductions, and the creation of atmosphere. In the context of tourism, existential experience involves personal or intersubjective feelings activated by the liminal process of tourist activities (Wang, 1999). Similarly, the audience can perceive the implicit concept of the authentic experience activated by the live streaming video. Taking *BoBo Chicken Seasoning* as an example, when the delicious food is served on the table, the audience seems to be in front of the table and smell the fragrance from the exclusive seasoning, even though they are actually thousands of miles away.

In addition, the interview guide was evaluated beforehand to avoid ethical issues, such as sexual discrimination, age discrimination, and income discrimination (Maison, 2019). The interviews were audio recorded to maintain the details. Then, the audio files were fed an online automated speech recognition (ASR) engine, iFlytek (https://global.xfyun.cn/), which is enabled by the AI technology to transcribe Chinese speech into text. Although the transcription machine has a very high recognition precision, the textual outcomes were double checked and corrected by a human transcription assistant to increase the accuracy. Finally, the output texts were entered into NVivo 11.0 for further qualitative analysis (Bazeley, 2013).

The interviews were conducted between January 2022 and March 2022. Before each interview, the participants were informed of the purpose of the study and were guaranteed the academic usage of the data and their personal privacy. The participants were told not to worry about right or wrong answers, but to freely contribute their own feelings, ideas, and opinions. Most interviews with college student participants were conducted face-to-face, while others with company employee participants were conducted *via* WeChat voice because of the different social distancing measures related to COVID-19. Each participant allowed the interviewer to record the audio. Each participant received 10 CNY as an incentive. All interviews lasted at least 40 min, wherein the longest interview lasted more than 1 h.

#### 4.2.3. Analysis

The grounded theory allows data collection and data analysis to occur simultaneously. In this study, the first batch of interviews was conducted with 16 recruited participants. After following the process of open coding, theoretical coding, and selective coding, several extracted new themes and topics were appended into the interview guide. The second batch of interviews was conducted based on the updated version of the guide. This time, seven participants were recruited, and after that, the study achieved the theoretical saturation, which meant that no additional data were detected that contributed to the development of the final categories. The COVID-19 epidemic had made interviews slightly more difficult. However, we realized that we had captured sufficient data for writing theory. Finally, twenty-three interviews were conducted in total; nearly 1,000 min of audio recording were made and 184,900 Chinese characters were accumulated.

The main purpose of the grounded theory approach was to generate a descriptive framework of the live commerce authenticity. The present study followed a four-stage procedure recommended by Glaser and Strauss (1967), Strauss and Corbin (1998), and Mahrous and Kortam (2012), including “open coding,” “theoretical coding,” “selective coding,” and “writing theory.” Using the “open coding” procedure, the data were identified and labeled as concepts. Combining with the authenticity and live commerce literature, the concepts and propositions extracted in the procedure of “open coding” were integrated into a theoretical framework. After conducting two batch of interviews, the iteration between data and concepts ended when “theoretical saturation” was reached. In the stage of “selective coding,” only those concepts associated with the core categories were selected to propose an abstract theory of live commerce authenticity. Finally, the categories were validated by comparing them with the concepts obtained through literature. The new theory were proposed to explain the concepts and their relationships in a logically theoretical framework.

## 5. Results and findings

The study followed a four-step paradigm of grounded theory: open coding, theoretical coding, selective coding, and writing theory. The open coding step was performed independently by one researcher and one trained college student. The two code files were merged, and after several discussions, the final code outcomes were used in the subsequent three steps, which were operated by the authors together.

### 5.1. Open coding

The textual contents were input into NVivo 11.0 software for open coding. The codes were labeled in Chinese for the convenience of subsequent processing. There were many miscellaneous codes at the beginning. In this step, categories need to be identified (Glaser and Strauss, 1967; Strauss and Corbin, 1998). The category is the conceptual element of a theory. Twenty-one categories were identified and sorted alphabetically, as shown in Table 2. Three codes and original interview texts were selected as examples for each category.

**Table 2:** Examples of 21 categories identified by open coding (in alphabetical order).

**Category**	**Concept**	**Original representative text**
Actualization	Affordability	… The products sold in live streaming are not expensive and I can afford them.
	Convenience	… There is no barrier to placing an order while I watch the live streaming.
	Practicability	… I find that the products recommended in live streaming are very practicable and useful.
Empathy	Empathy	… I can feel the difficulty XXX faces because my parents have experienced the same.
	Empathy	… The most delicious food comes from the most organic environment.
	Empathy	… COVID-19 caused poor sales, especially for the farmers.
Enjoyment	Enjoyment	… Yes. I meant it is so healing when you watch live streaming.
	Interesting	… XXX's videos are always interesting.
	Flow Experience	… The time passes so fast when I watch live streaming.
Experiential Feeling	Cultural Experience	… I like the cultural characteristics of the *Blue Calico*.
	Local Customs	… Through the streamer's lens, I seem to feel the color, the landscape, and even the smell
	and Practices	of Western Hunan.
	Visual Aesthetics	… In fact, the streamer's scene is not rough. Conversely, many steamers have high
		photography skills.
Familiarity	Humor	… XXX is interesting. Every time I laughed loudly.
	Neighborhood	… XXX looks like a neighbor in my hometown.
	Ordinariness	… XXX is an ordinary person.
Fan Effect	Attractiveness	… XXX is so beautiful that I like her very much.
	Follow	… I followed XXX's account, and now I watch her updates everyday. I learned more
		from her about how to take care of potted plants.
	Herd Effect	… XXX has millions of fans. I think she is the best.
Induced Demand	Impulsive Purchase	… Yes, I will place an order if I feel good.
	Supportive Purchase	… XXX truly worked hard. I need to help her.
	Trial Purchase	… The price is not high. Why not have a try? … They will be sent to my home.
Interaction	Interaction	… When XXX is on her streaming, there are so many users clicking *like*.
	Interaction	… The streamer often responds to questions and comments from fans in time.
	Interaction	… The details of the product will be presented on the screen which is easy for placing orders.
Intuitiveness	Intuitiveness	… It is easier for you to operate by following the live streaming than by the instructions.
	Intuitiveness	… The products are there. The effects are there, too.
	Intuitiveness	… Jiaqi Li (Austin Li) will show you the color after using. I know the difference and what I will choose.
Peculiarity	Cultural Diversity	… For example, one streamer I remember demonstrated one local food named XXX, which
		has a cultural story.
	Differentiation	… I agree with you that many streamers try to capture the audience's eye by differential contents.
	Individualization	… I like watching something different.
Physically	Recollection	… I lived that way when I was a child. It is familiar to me.
Experiential	Remembrance	… I miss the past time so much.
Simulation	Restoration	… XXX restores the atmosphere of the food. That is where it must be.
Recognition	Childhood Memories	… I recognized the scene. It is so familiar to me. I lived there until I left for college.
	Memory	… I remember XXX. She is the streamer of the *BoBo Chicken Seanoning*.
	Slogan	… “Buy it! Buy it!” [laugh] Jiaqi Li (Austin Li)'s slogan seems to have magical power.
Rich Information	Details	… XXX demonstrates the product from inside to outside. It is better than reading instructions
		on *Moubao* [referring to Taobao].
	Space-Time Advantage	… Yes. I know what you mean. Live streaming provides me the feeling of space and time.
	Space-Time Advantage	… You can follow the camera to move in the scene.
Sense of Involvement	Activity	… I collected the posts of XXX and when I wanted to cook I always remembered to find them
		and follow the steps.
	Cultural Movement	… I was interested in the local ceremony for Chinese New Year.
	Participation	… One day, XXX took us to catch his hens in the hills. Can you image that his hens were
		standing on the trees?
Sense of Reality	Illusion of Reallife	… XXX shows me her ordinary life. I know how she improves the seasoning.
	Illusion of Reallife	… XXX is somehow like a neighbor living near my home.
	Reality	… Everything in the lens seems real.
Social Sharing	Buy Together	… My friend and I planed to travel to Tibet because we watched the video of Zhen Ding.
	Discussion	… My friend and I once wanted to buy organic oranges. She recommended that I watch XXX.
	Forward	… I started to follow the XXX's account because my sister forwarded it to me.
Streamer	Celebrity	… I know Weiya (Viya). She is a charitable celebrity who has promoted many agricultural products
Image Building		for her hometown.
	Localization	… I like XXX's way of talking. She is the women who belongs to the village.
	Simplicity	… XXX gave me an impression of simplicity and purity.
Trustworthiness	Expertise	… It's amazing! I now know how to deal with the situation I have met before.
	Source Credibility	… I saw the harvest scenes and learned how to tell the good from the bad.
	Source Credibility	… I once bought pomegranates. … The streamer showed me the pomegranates. … They looked
		so fresh and mouth-watering.
Vicarious Feeling	Admiration	… When the food was served, my mouth was watering.
	Imitation	… I learned how to cook with *Peach Sister in Sichuan* several times.
	Local Accent	… People who live there have his accent.
Visualization	Visualization	… You know, consumers prefer to truly see.
	Visualization	… To me, the biggest benefit of live streaming is that I know what I will get.
	Visualization	… The size of the picture is not always the same as the real thing. … I employ situational information
		to judge the size of the product [via live streaming].
Telepresence	Avatar Effect	… XXX will change his demonstration procedure by replying to our audience.
	Community	… XXX has so many fans that we all feel like we are in one community. You can see them and feel
		them there.
	Presence Feeling	… I seemed to be at the site that time when the music was playing.

### 5.2. Theoretical coding

The second step of the analysis was theoretical coding (Glaser and Strauss, 1967). The concepts and propositions that emerged during open coding were reassembled to form a theoretical framework. Because of the inconvenience caused by COVID-19, the study adjusted the iterative process of further data collection and analysis to a two-batch process (Strauss and Corbin, 1998). In fact, it proved that such an adjustment also achieves category saturation rapidly. Table 3 lists the core categories and the corresponding connotation associated with authenticity in live commerce. By reassembling the relationship of the twenty-one categories, six core categories were induced, that is, “opinion leader building,” “affective commitment,” “product commitment,” “enhanced visual experience,” “telepresence experience,” and “shopping-related category.”

**Table 3:** The core categories and connotations associated with authenticity.

**Core category**	**Category**	**Connotation of category**
Opinion Leader	Streamer Image Building	Authenticity in live streaming will help the streamer build a more positive image.
Building	Recognition	The content of the scene is easy to form a memory in the subconscious.
	Social Sharing	The streamer's authenticity makes the live streaming more life-featured and promotes the
		sharing by the audience.
	Fan Effect	The streamer with authenticity is more popular; the audience will buy the products because
		they are the fan of the streamer.
Product	Intuitiveness	The features of the product will be clearer by presenting in the context of the application *via*
Commitment		live streaming.
	Rich Information	Through authentic product settings, the streamer enriches products' using scenarios and usages.
	Trustworthiness	The authenticity of products improves the audience's sense of trustworthiness during
		live streaming.
Affective	Enjoyment	There are more viewers of authentic content and users have a long retention time.
Commitment	Empathy	The authentic image and expression of the streamer help to form an intimate relationship.
	Vicarious Feeling	The expression of the streamer and the arrangement of the scene are close to life, which
		makes the audience replace themselves into the role and resonate more easily.
	Familiarity	The authenticity of the streamer makes the audience feel more familiar.
Enhanced Visual	Visualization	The visual simulation of offline scenarios makes up for the lack of trust in online consumption.
Experience	Peculiarity	Authenticity improves heterogeneity to help the streamers form their own styles.
	Experiential Feeling	The authenticity in the live streaming attracts the audience and makes them feel better and
		less bored.
Telepresence	Interaction	Live streaming provides intensive interaction among streamers and participants.
Experience	Physically	The authentic simulation of the physical scene helps the audience recollect and restore
	Experiential Simulation	their memories of the past life.
	Telepresence	The audience is immersed through the authenticity of the scene.
	Sense of Involvement	The simulation of scenes makes the audience feel more involved through the reproduction
		of real-life scenes, improving the intention of participation.
	Sense of Reality	Authentic scenes shorten the psychological distance between the streamer and the audience,
		making them feel that the product and the steamer are more realistic.
Shopping-Related	Actualization	Shopping *via* live streaming is easy to complete and the consumers are well protected.
Category	Induced Demand	The authentic settings grasp the audience's pain points to create demand.

### 5.3. Selective coding

**Figure 1:**
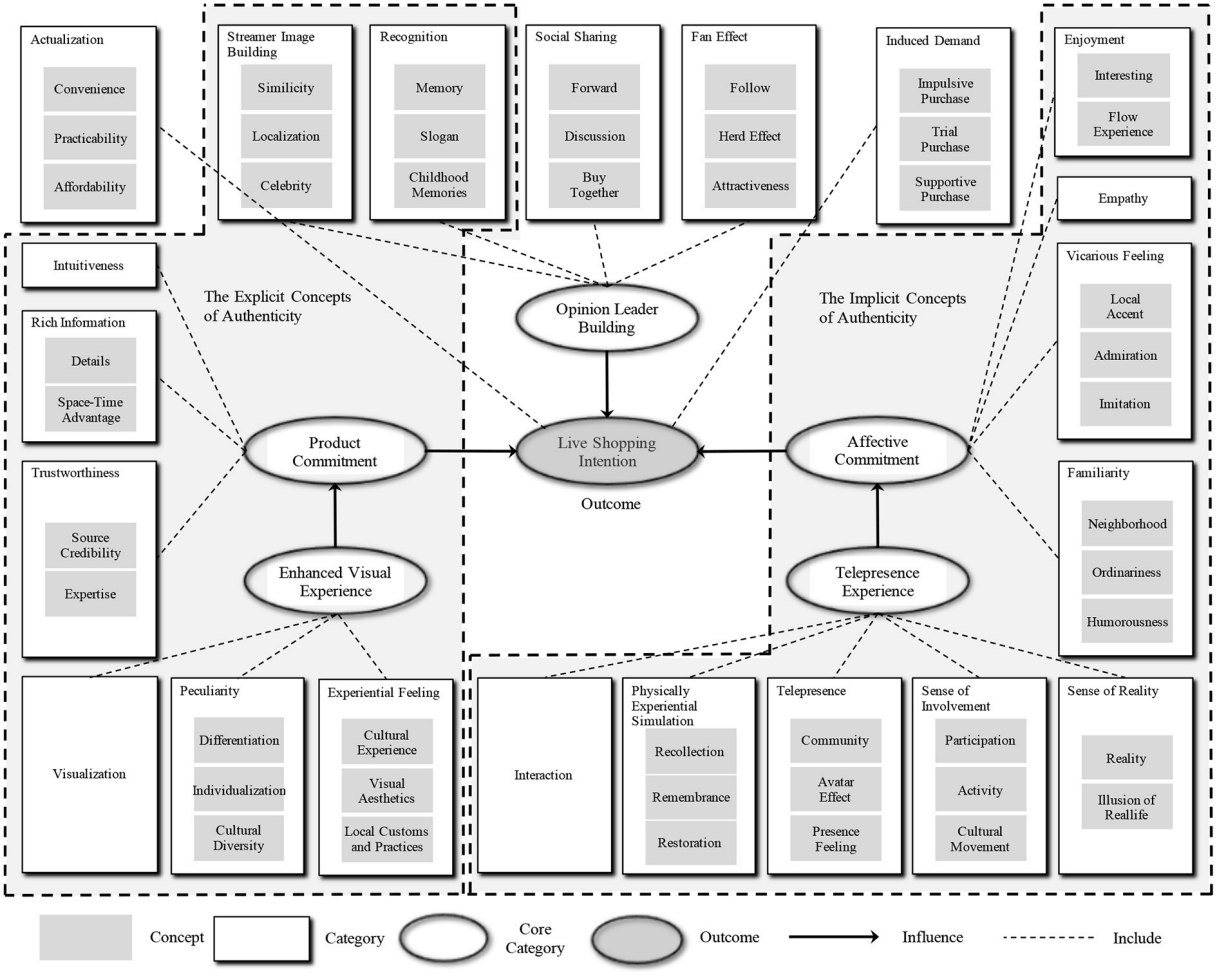
The conceptual framework depicts a grounded theory of the authenticity in live commerce and its impacts on participants' purchase behaviors.

As our study focused on the authenticity during live streaming and its impact on participants' shopping behaviors, the next step was to delimit coding to only those concepts that relate to the core categories. The theory was abstracted to the higher-level framework (shown in Figure 1) (Glaser and Strauss, 1967). The framework identified three indirect antecedents of live shopping intention: “opinion leader building,” “product commitment,” and “affective commitment,” as well as two direct antecedents: “enhanced visual experience” and “telepresence experience.”

### 5.4. Writing theory

In the final step, the emergent theory was written up, drawing the theoretical memos that had been written about each category during coding (Glaser and Strauss, 1967; Glaser, 1998; Strauss and Corbin, 1998). Inspired by the conventional classification of the authenticity, object authenticity and existential authenticity (Wang, 1999; Mody and Hanks, 2019), the explicit concept and implicit concepts of authenticity in live commerce were identified (referring to the two dashed frames in Figure 1).

#### 5.4.1. The effect of attractiveness

The streamer's attractiveness was mentioned to have positive effects on recruiting and retaining participants. These statements were in line with several references in the celebrity literature (Mathes and Kahn, 1975; Hamermesh, 2013; Slaton, 2013; Bascandziev and Harris, 2014; O'Connor and Gladstone, 2018). The streamer's attractiveness is considered to be associated with positive personal characteristics, e.g., self-esteem (Mathes and Kahn, 1975; Slaton, 2013), self-confidence (O'Connor and Gladstone, 2018), trustworthiness (Bascandziev and Harris, 2014), and being an opinion leader (Slaton, 2013). An attractive streamer will be more likely to and have an easier time gathering fans. For example, the famous streamers of Jiaqi Li (Austin Li), Weiya (Viya), and Ziqi Li, who have millions of fans, are all young, beautiful, and attractive. The herd effect among fans, followership, and attractiveness were reported to be the advantages of attractive streamers. Meanwhile, the attractive streamer will also be more likely to be the focal figure in the social situation. Being an attractive streamer will result in more forwarding, discussion, and collective shopping.

However, attractiveness also has disadvantages. *Homogeneous beauty* refers to the phenomenon of Internet celebrities all looking alike. They seem to be carved out of the same mold. They lack differentiation. “All happy families are alike; each unhappy family is unhappy in its own way,” Leo Tolstoy wrote in the his novel *Anna Karenina*. Similarly, the first part of the well-known saying can be applied to the celebrity attractiveness. The audience is used to the so-called *Internet celebrity face* with fair skin and good makeup. In contrast, the differentiated appearance and contents may become an alternative way to attract audiences.

To be an opinion leader, a streamer who does not have a good appearance needs to develop other competitive advantages. Authenticity is one option. According to the interviews, two categories of authenticity were identified in live commerce. As shown in Figure 1, the explicit concepts and implicit concepts are framed with dotted lines.

#### 5.4.2. Explicit concepts of authenticity

The explicit concepts of authenticity during live streaming refer to those categories of observable, sensible, and distinguishable cues attached to the streamer, the product, or the scene. Corresponding to object authenticity, the explicit concepts of authenticity convey the visual cues of physical features and cultural characteristics attached to the products. The streamer adopts a differentiated competition strategy to make a strong impression on the audience. Part of the audience living in modern cities may be interested in and yearn for the simple pastoral life. Other parts of the audience may be interested in local cultural affairs. They are the new learners of unknown or unfamiliar things. Hence, the streamers have the opportunity to be opinion leaders and build their celebrity images by demonstrating authentic information to the audience. To make a clear and deep impression, some streamers deliberately design recognizable slogans, colors, clothes, and labels. In live commerce, so many streamers compete for consumers' attention. They need to capture consumers' mindsets. Authentic content is a good anchor to encourage consumers to remember, recognize, and recall the streamer.

#### 5.4.3. Enhanced visual experience

Live streaming provides the streamer with a remote audio-visual channel to convey authentic cues. The space-time advantages visualize the demonstration. In the conventional e-commerce, consumers can only read the online descriptions and pictures of the products. Sellers often employ professional photographers to take high-quality pictures to attract consumers. Although most consumers prefer good-looking pictures, they do not have actual and credible information before they receive the products. Some e-commerce platforms provide buyers with the ability to upload the *Buyers' Show* pictures, which are more actual but may be less beautiful than the *Sellers' Show*. In live commerce, consumers watch the live streaming, and they learn the shape, size, color, and even the function of the actual product. A qualified streamer takes visual advantage to present authentic characteristics, e.g., local settings, tastes, environments, and histories. These cues are easier to integrate within the live streaming framework.

In addition, the live streaming is also a suitable approach to demonstrate uniqueness and increase consumers' experiential feelings. Most of the time, authentic objects and contents mean differentiation, individualization, and cultural diversity. Many informants admitted that they prefer seeking something different. Live streaming provides direct visual stimulation. Meanwhile, authenticity is also an experiential feeling. Some streamers demonstrate cultural diversity in an aesthetic way, e.g., Ziqi Li. They may have a professional production team. Some informants said that they enjoyed watching and learning the local customs and practices through the lens of live streaming. Therefore, the explicit concepts of authenticity are enhanced visual experiences of the audience, subsequently resulting in their product commitment.

#### 5.4.4. Product commitment

The audience can easily recognize the authenticity from the product descriptions, the local and cultural design of the products, and the knowledge of local customs and practices. However, consumers cannot obtain detailed information for their decision-making in conventional e-commerce. Live streaming establishes a space-time scenario for the streamer to demonstrate products from various aspects. Consumers intuitively learn the basic characteristics, features, functions, and even the effects. One informant mentioned that “Jiaqi Li (Austin Li) will show you the color after using.” Many cosmetic consumers worry about the actual effect on themselves after using the product, even if the advertisement gives a wonderful display. They need to know the details before placing an order. Alternatively, Taobao consumers are used to inquiring about product details from online customer services before shopping. One informant said this is time-consuming. They prefer to learn, feel, and anticipate by following the camera during live streaming. Trustworthiness is the outcome of the expertise and source credibility of the authenticity in live commerce. Therefore, the consumers will increase their product commitment because of the explicit authentic cues.

#### 5.4.5. Implicit concepts of authenticity

The audience can also perceive the authenticity from scene settings. The authentic scenes and atmosphere involve the audience through the environmental settings attached to the products. Corresponding to existential authenticity, the implicit concepts of authenticity bring the audience into an elaborate atmosphere to connect with the authentic environments, which were classified as the “telepresence experience” in this study.

#### 5.4.6. Telepresence experience

In this study, five categories of telepresence experience were identified, i.e., interaction, physically experiential simulation, telepresence, sense of involvement, and sense of reality. Referring to Wang (1999), existential authenticity may not be associated with the toured objects directly when tourists engage in activities of camping or wilderness solitude. The explanation is that they search for their authentic selves, which means their experiences and feeling are connected with the authentic environment. Sometimes, part of the audience also desires that the experience is connected with the authentic scene presented by the live streaming.

Explicit concepts of authenticity were more easily perceived by the informants than implicit concepts. The informants had different levels of understanding of the academic term of “authenticity.” We need to explore the dimension of implicit concepts with the help of authenticity theories (MacCannell, 1973; Wang, 1999; Zatori et al., 2018). Telepresence experience refers to the participant's existential experience in the remote scenes. Live streaming is mainly developed for unidirectional one-to-many broadcasting most of the time. However, live commerce apps also provide bidirectional interactions between the streamer and the audience. From the parasocial perspective, a live commerce app acts as a medium between the streamer and the audience. Many participants may feel the streamer knows them and notices their responses. Some informants stated that the streamer often responded to questions and comments from fans in real time. Other categories associated with telepresence include the avatar effect, community, and feeling of presence. One informant stated that the streamer once changed the demonstration procedure by replying to the audience's questions, making her feel like she had remote control of the demonstration. Furthermore, there are so many fans in the live streaming room that the participants think they are taking part in a community and seem to meet and feel each other. Some informants stated that their feeling of being present comes from the experience and memory of the actual scenes. Sometimes, they seemed to be at the site when the specific music was playing.

Some informants perceived the real-life atmosphere through the lens of live streaming videos. They said that they had a sense of reality when they followed the procedure of the actual activities, e.g., cooking or traveling. They felt that the scenes seemed to be real. The illusion was so real that one informant said, “XXX is somehow like a neighbor living near my home.” Some informants perceived involvement and engagement during live streaming. They felt that they took part in the activity presented by the streamer. One informant told an interesting story that the streamer took them to catch his hens in the hills to prove that his hens were all organic. Some informants stated that they felt involved in the cultural movement during the local ceremony for Chinese New Year ceremonies.

In addition, some streamers often simulate the physical consumption scenes to attract audiences. Some middle-aged consumers felt as if the clock was turned to the past. In these cases, the remote scenes were never artificial; they were very real and familiar to the informants. Live streaming helps them recall and restore sensory memories of their past lives. Some streamers established the final effect scene of the product usage and invited participants to join the trial activity, evoking the presence perception of the participants.

In summary, although the audience cannot actually took part in the remote activities, they were brought into a simulated and interactive environment, making them feel involved and engaged in the actual activities. The telepresence experience is not evoked by explicit authenticity but implicitly generates and exists in the hearts of the audience. The enhanced visual experience leads to consumers' product commitment, and the telepresence experience leads to consumers' affective commitment.

#### 5.4.7. Affective commitment

Different from the explicit concepts of authenticity, the implicit concepts of authenticity is highly related to consumers' affective commitment. In this study, four core categories were identified as being associated with affective commitment, i.e., enjoyment, empathy, vicarious feelings, and familiarity. Authentic scenes and atmosphere may evoke the audience's interest and flow experience, making them feel enjoyment. Meanwhile, some informants stated that they could feel the difficulty the streamer faced and that COVID-19 enhanced their feelings of empathy. In addition, the audience feels an emotional commitment from vicarious feelings. The streamer's local accent evoked some informants' emotions of substitution. The audience felt familiar when they experienced a humorous, or ordinary-looking streamer. Therefore, although the implicit concepts of authenticity do not transfer authentic cues to consumers, they evoke their affective commitment, which will, in turn, impact their shopping decision-making.

#### 5.4.8. Shopping-related categories

Finally, the core categories of actualization and induced demand are related to shopping behavior. Most consumers participate in live commerce because of utilitarian motivations. One informant said that he could afford the product promoted in the live commerce because it is cheap. Another informant stated that shopping *via* the live streaming app (Douyin) was convenient and easy to operate. In addition, the products recommended in streaming are practical and useful. From the perspective of emotional motivations, affective commitment will induce consumers to make purchases. One informant reported that he placed an order because he was persuaded to have a try. One informant also stated that she often made impulsive purchases if she felt good. Another informant stated that she wanted to support the streamer because she knew the streamer worked hard, in a type of supportive purchase.

## 6. Conclusions and implications

The qualitative exploration in this paper provides a deeper understanding of the influence of streamer attractiveness and authenticity on consumers' purchase intention in live commerce. These insights partially answered the questions posed at the beginning of this paper. Using a qualitative approach, we found that attractiveness indeed has an effect. However, most celebrities tend to present a “homogeneous beauty” to the audience, resulting in “aesthetic fatigue” (Mathes and Kahn, 1975; Hamermesh, 2013). If the streamer has no advantage in appearance, (s)he may try to implement the authenticity strategy. Authenticity during live streaming can be perceived by the audience, although most people have different levels of understanding of authenticity. The authenticity strategy proves to be a differentiated competitive strategy for both attractive and nonattractive streamers to build an image as an opinion leader.

After interviewing 23 informants, we achieved 21 initial categories and reached the saturation. We then classified them into six core categories: “opinion leader building,” “product commitment,” “affective commitment,” “enhanced visual experience,” “telepresence experience,” and “shopping-related category.” Although qualitative research approaches are questioned for their subjective bias, the present study still contributes to the live commerce literature by identifying two concepts of authenticity: explicit and implicit concepts of authenticity. Furthermore, by resembling the relationship among the core categories, we explored two influence paths of authenticity on consumers' live shopping behaviors. These findings were aligned with the conventional authenticity literature: object authenticity and existential authenticity (MacCannell, 1973; Wang, 1999; Boukas, 2012). Most informants recruited in this study easily distinguish the explicit concepts of authenticity because these so-called authentic cues are observable and better communicated *via* live streaming. Live streaming enhances consumers' visual experience by transferring cultural and individual information to consumers. These cues seem to be intuitive, professional, credible, and recognizable. They are connected to consumers' product commitment. That is, the explicitly authentic cues transferred *via* live streaming will enhance consumers' visual experience and increase their trustworthiness, which subsequently leads to consumers' product commitment.

However, most informants recruited in this study rarely recognize the implicit concepts of authenticity. According to the authenticity theory, object authenticity refers to the objective, external and cultural elements and scenes attached to the products, whereas existential authenticity refers to the subjective, internal, and affective experience and engagement linked to the consumers themselves (Wang, 1999). Although many informants reported perceptions of involvement, presence, and participation, most of them did not realize the authenticity of the streaming. This is the reason why we classified authenticity into two large categories of explicit concepts and implicit concepts. Affective commitment is generated from the consumers' telepresence experience. In turn, the telepresence experience is generated when the consumers interact with the streamer and the community, participate in promotion activities and cultural movements, and substitute themselves into remote local life. Moreover, implicit concepts of authenticity are associated with strong emotions, e.g., recollection, remembrance, and restoration, which result in familiarity, empathy, and enjoyment with the streamer.

Finally, this study also identified two shopping-related behaviors from the two sides of authenticity. From the utilitarian side, convenience, practicability, and affordability are associated with consumers' shopping intention when they receive explicitly authentic cues *via* live streaming. From the emotional side, the implicit concepts of authenticity are associated with induced demand, e.g., leading to consumers' impulsive purchases, trail purchases, and supportive purchases. In summary, this study achieved several theoretical and practical implications.

This study contributes to the authenticity literature by identifying two categories of authenticity in live commerce, i.e., explicit concepts of authenticity and implicit concepts of authenticity. As live commerce is an emerging marketing phenomenon, there is still a lack of relevant studies. Live streaming technology enables marketing tools to present authentic cues from remote scenes. However, how consumers perceive authenticity *via* live streaming is still unknown. This study conducted an initial exploratory study by employing a qualitative approach. Although the qualitative approach is often questioned for its subjective bias, the outcome of the several detailed categories associated with both explicit and implicit concepts of authenticity may be useful and may inspire future research.

With the rapid development of live commerce, there are increasing numbers of online streamers. How are consumers attracted to them, and how are products promoted by them? This is a crucial problem for live commerce and live marketing. Beautiful streamers can take advantage of an attractive appearance to build a celebrity image and recruit fans. However, this is not the only way to succeed. Authentic attractiveness may be beneficial for both attractive and nonattractive streamers. Moreover, this study revealed that product commitment and affective commitment play important roles in consumers' live shopping decision-making. Although consumers can more easily perceive explicitly authentic cues than implicitly authentic experiences, this study suggested that the latter may be more effective in inducing shopping behaviors.

## 7. Limitations and suggestions for future research

Because the live commerce is more popular in China than in other countries, this study only focused on Chinese consumers. The informants recruited were all Chinese people, so the outcomes were highly related to Chinese culture. The outcomes need to be generalized to other countries. Second, because of the COVID-19 epidemic, the convenience sampling procedure was difficult. The study adapted the iteration procedure of grounded theory by a two-batch procedure to reach the theoretical saturation. Finally, the results were not verified by the quantitative approaches of this study. In the future, the authenticity of live commerce will be more carefully and rigorously identified in different cultural contexts, and more quantitative studies will be conducted to verify the results.

## Data availability statement

The original contributions presented in the study are included in the article/supplementary material, further inquiries can be directed to the corresponding author.

## Author contributions

JS and AZ: conceptualization and methodology. JS, HD, and AZ: writing and analysis. JS: investigation. HD and AZ: validation and visualization. All authors have read and agreed to the published version of the manuscript.

## Funding

This research was funded by the National Natural Science Foundation of China (Grant No. 71971198), Philosophy and Social Science Planning Foundation of Zhejiang Province (Grant No. 21NDJC080YB). This research was also supported by the Special Project for International Student Education & Management Service of Zhejiang Gongshang University and the Teaching Platform Project of Zhejiang Province.

## Conflict of interest

The authors declare that the research was conducted in the absence of any commercial or financial relationships that could be construed as a potential conflict of interest.

## Publisher's note

All claims expressed in this article are solely those of the authors and do not necessarily represent those of their affiliated organizations, or those of the publisher, the editors and the reviewers. Any product that may be evaluated in this article, or claim that may be made by its manufacturer, is not guaranteed or endorsed by the publisher.
